# Epithelioid hemangioendothelioma of the pancreas presented as massive hematemesis

**DOI:** 10.1016/j.ijscr.2019.10.016

**Published:** 2019-10-18

**Authors:** Sardar Hassan Arif, Ayad Ahmad Mohammed

**Affiliations:** Department of Surgery, College of Medicine, University of Duhok, Kurdistan Region, Iraq

**Keywords:** Hemangioendothelioma, Epithelioid hemangioendothelioma, Whipple’s operation, Pancreaticoduodenectomy, Upper GIT bleeding, Hematemesis

## Abstract

**Introduction:**

Hemangioendotheliomas are vascular tumors characterized by the presence of an “epithelioid” or “histiocytoid” endothelial cell. They consist of blood filled spaces mixed with cellular areas that mostly occur in the liver, but other organs can be affected. These lesions cause a wide spectrum of clinical presentations depending on the organ involved. Complete surgical excision is the primary form of treatment; they tend to recur after surgery in some cases.

**Presentation of case:**

A-45-year old lady presented with 2 attacks of massive hematemesis. Upper GIT endoscopy was done which showed a vascular tumor arising from the duodenal ampulla, biopsy was not taken because of the risk of bleeding.

Laparotomy and duodenotomy were performed, there was a soft irregular 3 cm mass with areas of hemorrhage arising from the duodenum, complete excision was done and frozen section examination showed a benign lesion.

Two months later the patient presented with melena and anemia. CT-scan of the abdomen showed a mass at the head of pancreas. Second surgery was done and during surgery a firm mass was found in the region of the head of pancreas, pancreaticoduodenectomy was done and the sample was sent for histopathological examination which showed an epithelioid hemangioendothelioma of the head of the pancreas.

**Discussion & conclusion:**

The treatment strategy should be tailored to the individual patient, the clinical presentation, and the rate of progression of the tumor. The natural history of the disease is still poorly understood and there is no uniform treatment form for such tumors.

## Highlights

• Hemangioendothelioma is vascular tumors characterized by the presence of an “epithelioid” or “histiocytoid” endothelial cell.
• The majority of the tumors affects the liver.
• The natural history of the disease is still poorly understood and there is no uniform treatment form for such tumors.

## 1 Introduction

Hemangioendothelioma (HE) is a unique subtype of a group of vascular tumors that is characterized by characterized by the presence of an “epithelioid” or “histiocytoid” endothelial cell. The term HE was first described as a distinct tumor by Weiss and Enzinger in 1982, when they defined this tumor as soft tissue vascular lesions originating from the endothelium and they have a clinical behavior in-between benign hemangiomas and angiosarcomas [[1], [2], [3]].

These types of tumors tend to affect females mostly with female: male ration of 3:2, and mostly affect those more than 40 years, although their occurrence at younger age groups had been reported [3].

The lesions consist of blood filled spaces mixed with cellular areas. These vascular tumors can occur in various organs in the body such as the liver, skin, spleen, salivary glands, and some other anatomical sites. The tumors stain positively with one of the vascular markers on immunohistochemistry such as factor VIII related antigen, CD31 and/or CD34 [2,4,5].

These lesions cause a wide spectrum of clinical presentations depending on the organ involved, cases have been reported presenting with abdomen pain, jaundice, and duodenal obstruction [6,7].

The work of this case report has been reported in line with the SCARE criteria [8].

### 1.1 Patient information

A-45-year old lady presented with melena for 20 days, she had two attacks of massive hematemesis before admission. The patient had no history of any medication intake before this presentation. The patient had history of renal cell carcinoma which was operated by right nephrectomy before 10 years.

#### 1.1.1 Clinical findings

At admission the pulse rate was 120 beats/minutes and the blood pressure was 95/60 mm Hg. Resuscitation was done in the form of 2 peripheral intravenous lines and 2 liter of Ringer’s lactate solution, the patient received 9 units of fresh whole blood and 15 units of fresh frozen plasma.

#### 1.1.2 Diagnostic assessment

**Fig. 1:**
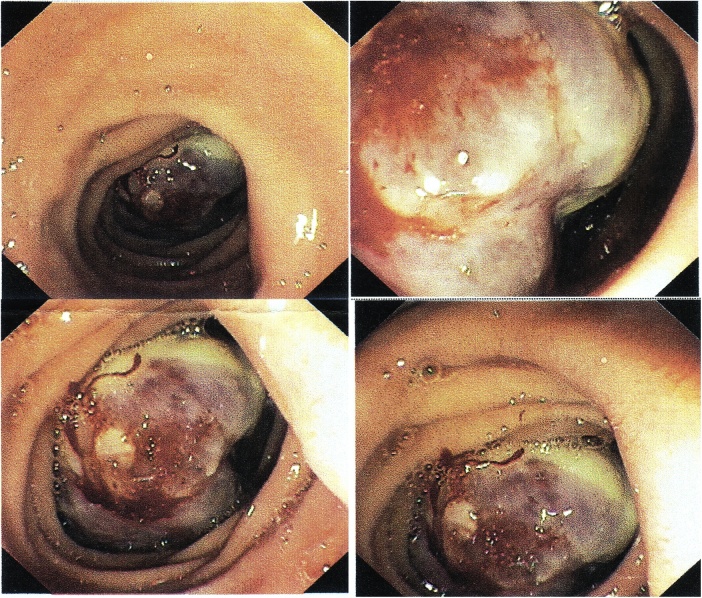
Endoscopic view showing an irregular hemorrhagic mass at the second part of the duodenum.

Upper GIT endoscopy was done, biopsy was not taken because of the risk of bleeding, Fig. 1.

#### 1.1.3 Therapeutic intervention

**Fig. 2:**
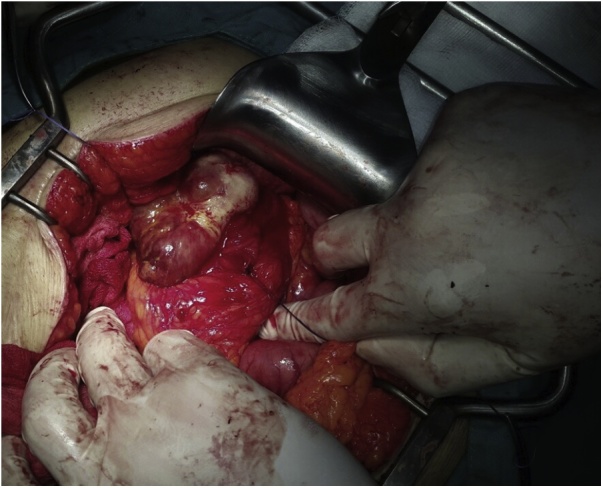
Intraoperative picture showing an irregular mass with hemorrhagic spots arising from the wall of the duodenum, after the lumen of the duodenum had been opened.

Decision for surgery was done, during laparotomy and duodenotomy, there was a soft irregular 3*5 cm mass with areas of hemorrhage arising from the wall of the duodenum, complete excision was done and sent for frozen section examination which showed a benign lesion, Fig. 2.

The duodenum was closed transversely and the patient was discharged in the 5^th^ postoperative day with no complications.

The histopathology report after the first surgery revealed a benign inflammatory lesion or a possibility of polyp.

Two months later she presented with melena and anemia. She received 6 units of blood.

**Fig. 3:**
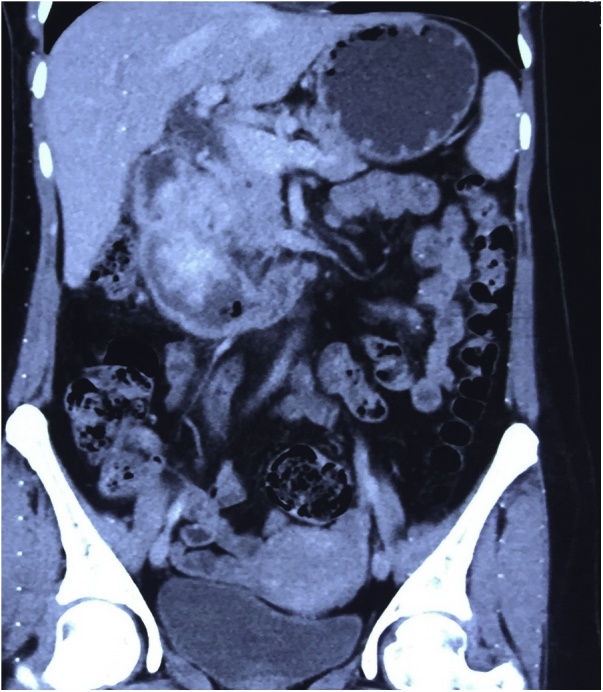
CT-scan of the abdomen showing an irregular mass with mixed enhancement arising from the region of the head of the pancreas.

Upper gastro-intestinal tract (GIT) endoscopy was done which showed recurrence of the mass, the patient was sent for CT-scan of the abdomen which showed a 7*4 cm mass with extension to the head of the pancreas, Fig. 3.

**Fig. 4:**
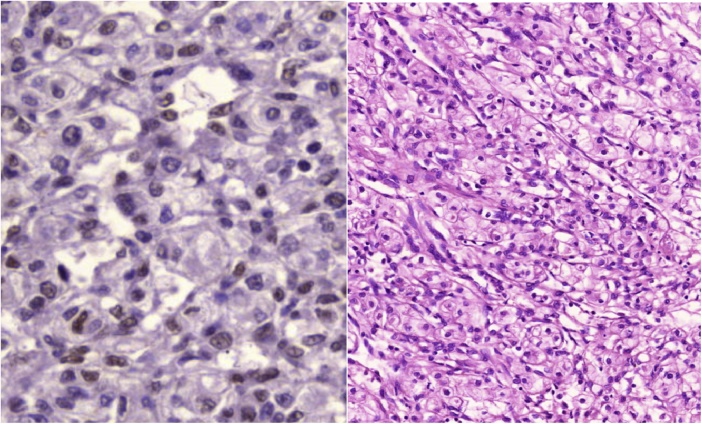
A microscopic view of the tumor showing the vascular spaces lined by endothelial cells with intervening epithelioid spindle cells, there is no evidence of nuclear atypia.

Decision for pancreaticoduodenectomy was done. During surgery a firm mass was found in the region of the head of pancreas found, pancreaticoduodenectomy was done and the sample was sent for histopathological examination which revealed a vascular mass lined by endothelial cells with intervening epithelioid spindle cells, there was no evidence of nuclear atypia, the tissue stained positively for CD 34, FL1, and pancreatin and stained negatively for CD117, S100, desmin and chromogranin A. These features were consistent of epithelioid hemangioendothelioma of the head of the pancreas, all the resection margins were free from the tumor, Fig. 4.

#### 1.1.4 Follow-up and outcomes

The patient was discharged 7 days after surgery with no complications. Follow up was done for 2 years after the surgery with no recurrence of the tumor.

## 2 Discussion

The majority of these tumors affect the liver which occur in around 70% of the cases, with right lobe involvement more than the left one and tend to be multifocal. Extrahepatic involvement is observed in the rest of the patients, the lung is the second most common organ that is involved, other organs also may be involved such as the lymph nodes, the bones, the peritoneum, and the diaphragm in order of decreasing frequency [3].

The tumor histological examination such as nuclear pleomorphism and mitotic rate don’t predict the biological behavior of the tumor [5].

Local recurrence is common which may be due to contiguous spread or multifocality of the disease, metastatic disease is extremely rare [4].

The treatment of such tumors depend on the size of the lesion, the location, and the presentation. Some lesions are treated with enucleation, others are treated with local or wide excision, systemic use of steroids and interferon have been used with different responses, in our case we didn’t use these form of treatment because the patient had emergency presentation, we performed complete surgical excision as a primary form of treatment [9,10].

The tumor mostly recurs locally, but in some rare occasions these tumors metastasize to other organs, the mediastinum, the retroperitoneal space, the myocardium, the pericardial membrane, the brain, the local lymph nodes draining the anatomical area of the primary tumor, the pancreas, and the uterus are some sites of distant metastatic disease that were reported by some authors [3].

Currently there is no uniform modality for treatment of these tumors, and all the therapeutic trials are directed for individual cases, local and international guidelines are required for the management of these tumors depending on the organ involved and the long term follow up of the registered cases.

## 3 Patient’s perspective

I thought that the mass in the pancreas is recurrence of my renal tumor, I am happy with the results of surgery and I will keep regular check up in the future.

## Sources of funding

None.

## Ethical approval

Ethical approval has been exempted by my institution for reporting this case.

## Consent

Written informed consent was obtained from the patient for publication of this case report and accompanying images.

## Author contribution

Dr Ayad Ahmad Mohammed and Dr Sardar Hassan Arif are the surgeons who performed the operation.

The concept of reporting the case, data recording, and drafting the work done by Dr Ayad Ahmad Mohammed and Dr Sardar Hassan Arif.

Dr Sardar Hassan Arif took the consent from the patient for publishing the case.

Final approval of the work to be published was done by Dr Ayad Ahmad Mohammed.

## Registration of research studies

This work is case report and there is no need of registration.

## Guarantor

Dr Ayad Ahmad Mohammed is guarantor for the work.

## Provenance and peer review

Not commissioned, externally peer-reviewed.

## Declaration of Competing Interest

The author has no conflicts of interest to declare.
